# Optogenetic Modulation and Multi-Electrode Analysis of Cerebellar Networks *In Vivo*

**DOI:** 10.1371/journal.pone.0105589

**Published:** 2014-08-21

**Authors:** Wolfgang Kruse, Martin Krause, Janna Aarse, Melanie D. Mark, Denise Manahan-Vaughan, Stefan Herlitze

**Affiliations:** 1 Department of Zoology and Neurobiology, Faculty for Biology and Biotechnology, Ruhr University Bochum, Bochum, Germany; 2 Department of Neurophysiology, Medical Faculty, Ruhr University Bochum, Bochum, Germany; 3 International Graduate School of Neuroscience, Ruhr University Bochum, Bochum, Germany; Tokyo Medical and Dental University, Japan

## Abstract

The firing patterns of cerebellar Purkinje cells (PCs), as the sole output of the cerebellar cortex, determine and tune motor behavior. PC firing is modulated by various inputs from different brain regions and by cell-types including granule cells (GCs), climbing fibers and inhibitory interneurons. To understand how signal integration in PCs occurs and how subtle changes in the modulation of PC firing lead to adjustment of motor behaviors, it is important to precisely record PC firing *in* *vivo* and to control modulatory pathways in a spatio-temporal manner. Combining optogenetic and multi-electrode approaches, we established a new method to integrate light-guides into a multi-electrode system. With this method we are able to variably position the light-guide in defined regions relative to the recording electrode with micrometer precision. We show that PC firing can be precisely monitored and modulated by light-activation of channelrhodopsin-2 (ChR2) expressed in PCs, GCs and interneurons. Thus, this method is ideally suited to investigate the spatio/temporal modulation of PCs in anesthetized and in behaving mice.

## Introduction

Purkinje cells (PCs) are the sole output of cerebellar cortex and are part of the computational network involved in motor planning and execution. PCs reveal a spontaneous firing rate [1] that is modulated during movement [2], [3]. Various anatomical structures contribute to the dendritic circuitry of PCs. Axons of molecular layer interneurons (MLI) (basket and stellate cells) traverse sagittally, while axons of granule cells (GCs) (parallel fibers) run coronally relative to the PC cell body and dendritic tree. The anatomical arrangement of MLI and GC axons in the PC layer has led to the beam hypothesis [4], [5], stating that signals generated by GCs are conveyed along the parallel fibers to activate beams of PCs. However, the functional input and output relationships of PCs have been difficult to elucidate despite the detailed anatomical information (for review see [6]). In the past, cerebellar function has been analyzed by various approaches including lesions, electrical stimulation or drug applications. However, these approaches lack cell type specificity. From these methods, only electrical stimulation allows for temporal precise interaction on the millisecond time scale, but it is difficult to affect multiple cells of a specific type simultaneously. To precisely investigate the underlying cerebellar micro-circuitry during movement *in* *vivo*, new methods for targeted stimulation of distinct cell classes have to be developed.

In recent years, optogenetic technologies have made new tools available to manipulate the activity of targeted neuronal populations *in* *vivo*. With light-activated proteins such as channelrhodopsin-2 (ChR2) [7]–[9], halorhodopsin (NpHR) [10] ARCH [11] or vertebrate opsins [9], [12], [13] it is now possible to activate, inhibit or modulate action potential firing in the time-scale of milliseconds *in* *vivo*, by application of light to the neuronal tissue. The expression of light-activated proteins can be restricted to specific neuronal populations using the Cre/loxP recombinase system in combination with cell-type specific promotors. For example, delivering an adeno-associated virus (AAV) that expresses a floxed opsin to a brain region with Cre-expressing cells will induce the expression of the opsin in a cell-type specific manner [14]. In our study we used different transgenic mouse lines, i.e. tgPcp2-cre, knock-in GAD2-cre and tgGAB6-cre, in combination with stereotactic injection of floxed virus in the cerebellar vermis to allow for selective expression of ChR2 in PCs, GABA-ergic MLIs and GCs, respectively.

Current optogenetic methods to control neuronal activity by light use light beams, which are directly applied on the exposed surface of the structure (e.g. [14]–[16]), or an optical fiber, which is inserted into the tissue (e.g. [17]–[19]). However, light scattering in the tissue makes the activation of deep structures difficult. For a more focused and spatially adjustable light delivery we now integrated an optical fiber into an established multi-electrode recording system [20]. With this technique we were able to illuminate at single or multiple sites in precisely defined and adjustable areas in the cerebellum, while recording with multiple, independently movable electrodes. The difference to other technical solutions where the light guide and the electrodes are directly spatially coupled [21]–[24] is that we now have the possibility to separate the optical fiber from the recording electrode. This allows the independent movement of light-guide and recording electrode to minimize damage to tissue during fiber movement.

In this study, we monitored the activity of PCs during application of light pulses through the optical fiber when ChR2 was expressed in PCs, MLIs or GCs. PCs responded with strong and immediate increases in simple spike rates when they were activated via ChR2 stimulation. Light-activation of MLIs by ChR2 leads to a complete block of PC spontaneous firing rates, whereas activation of GCs by ChR2 causes either a sustained increase or decrease in simple spike rates after prolonged GC activation. Thus, our study establishes a new method to precisely deliver light in spatially defined domains within the cerebellar cortex, and describes the functional modulation of PC firing by GCs and MLIs, when these cell-types are active.

## Results

### Spatial distribution of light activation with optical fiber

The goal of this study was to establish a method to precisely control the firing of different cerebellar cell-types in defined spatial domains using optogenetic techniques, while monitoring PC firing. PCs provide the sole output of the cerebellar cortex and were identified by the combined occurrence of simple and complex spikes (see Methods). To control neuronal firing of different cell-types in the cerebellar network, we used the channelrhodopsin variant ChR2(H134R) to selectively activate either PCs, MLIs or GCs by light.

In the first set of experiments, we injected double-floxed virus in the cerebellar vermis of tgPcp2-cre mice to specifically activate PCs by light. To attain almost equal ChR2 expression levels in the target area of the recordings, we waited two weeks after virus injection to allow for ample spreading of ChR2 expression before extracellular recordings and histological studies were performed. The floxed virus led to specific expression of mCherry tagged ChR2 in PCs of tgPcp2-cre mice.

**Figure 1:**
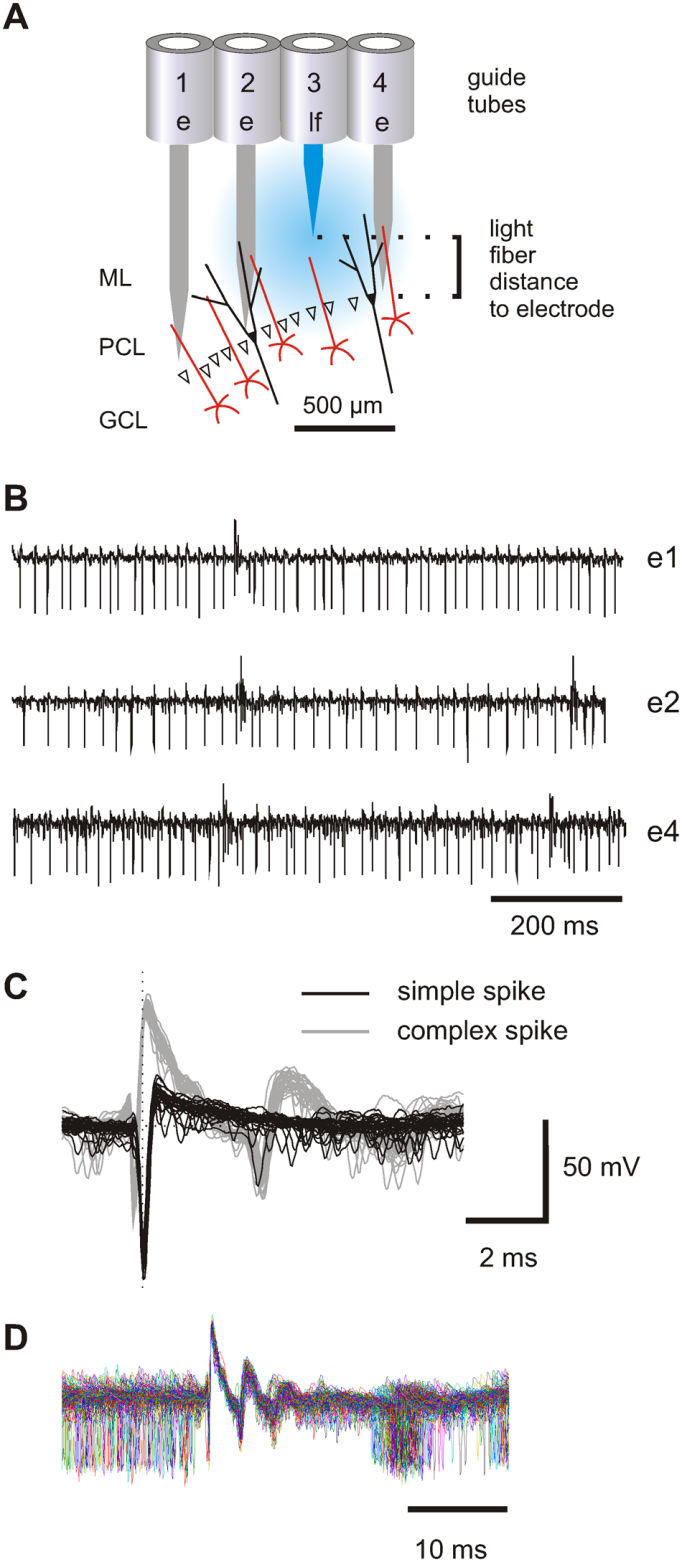
Simultaneous recordings from multiple PCs in the vermis of mice. (A) Schematic representation of relative positions of three electrodes and a single light guide assembled in a linear array during a recording and stimulation experiment in the cerebellum. The guide-tubes (1 to 4) are part of the multi-electrode system, which allows for independent vertical movement of each fiber. Guide-tubes 1, 2 and 4 each house an electrode while guide-tube 3 contains a light fiber. The electrodes are placed to enable recordings from the Purkinje cell layer, whereas the tip of the light-guide resides in the molecular layer. (ML: molecular layer, PCL: Purkinje cell layer, GCL: granular cell layer). (B) Simultaneous recording from three PCs with electrodes e1, e2 and e4. Complex spikes can be identified by upward deflection of the action potential. (C) Superposition of 30 simple spikes (black) and 30 complex spikes (gray) recorded from electrode e2. (D) Complex spike triggered superposition of raw traces (n = 295) shows that simple spikes do not occur for 15 ms after complex spikes. The simple spike pause indicates that simple and complex spikes are recorded from the same cell.

For the combination of extracellular recordings and focal light application, a 7-channel micromanipulator (Eckhorn System, Thomas Recording, Giessen, Germany) was adapted to house one or multiple glass fibers. The longitudinal position of the fibers can be controlled independently and with the same accuracy as of single electrodes (see Methods). A 473 nm laser beam was fed into the glass fiber resulting in a focal light beam surrounding the tip of the fiber (Fig. 1A and figure S1). As the headpiece of the multi-electrode system is exchangeable, either a linear array or a concentric configuration of the guide tubes was used. In the concentric configuration, the optical fiber was always placed in the central guide tube. The radial distance to the six neighboring electrodes was therefore fixed to 330 µm, which corresponds to the width of the guide tubes. In the linear array, the horizontal distance between electrodes and optical fiber was equivalent to multiples of 330 µm (Fig. 1A). The craniotomy for the recording electrodes was centered to the craniotomy of the previous virus injection. Electrodes and light guides were always driven vertically into the cerebellum, in parallel to the track of the pipette used previously for virus injection. As the virus was injected at multiple sites along the vertical track of the pipette between 500 and 2000 µm below the pia, we usually experienced little variation in the overall responsiveness of the PCs to light when the electrodes crossed PC layers at different depth.

Signals from up to six electrodes were recorded simultaneously. An example recording from three electrodes is shown in Fig. 1B. Recordings from PCs were identified by the combined appearance of simple spikes and complex spikes in the same signal (Fig. 1B&C). Thus, simple and complex spikes originate from a single PC when each complex spike is followed by a short pause (several ms, “climbing fiber pause”, [2], [25] in simple spike firing (Fig. 1D)). In this study, only cell activity from PCs was used for analysis.

**Figure 2:**
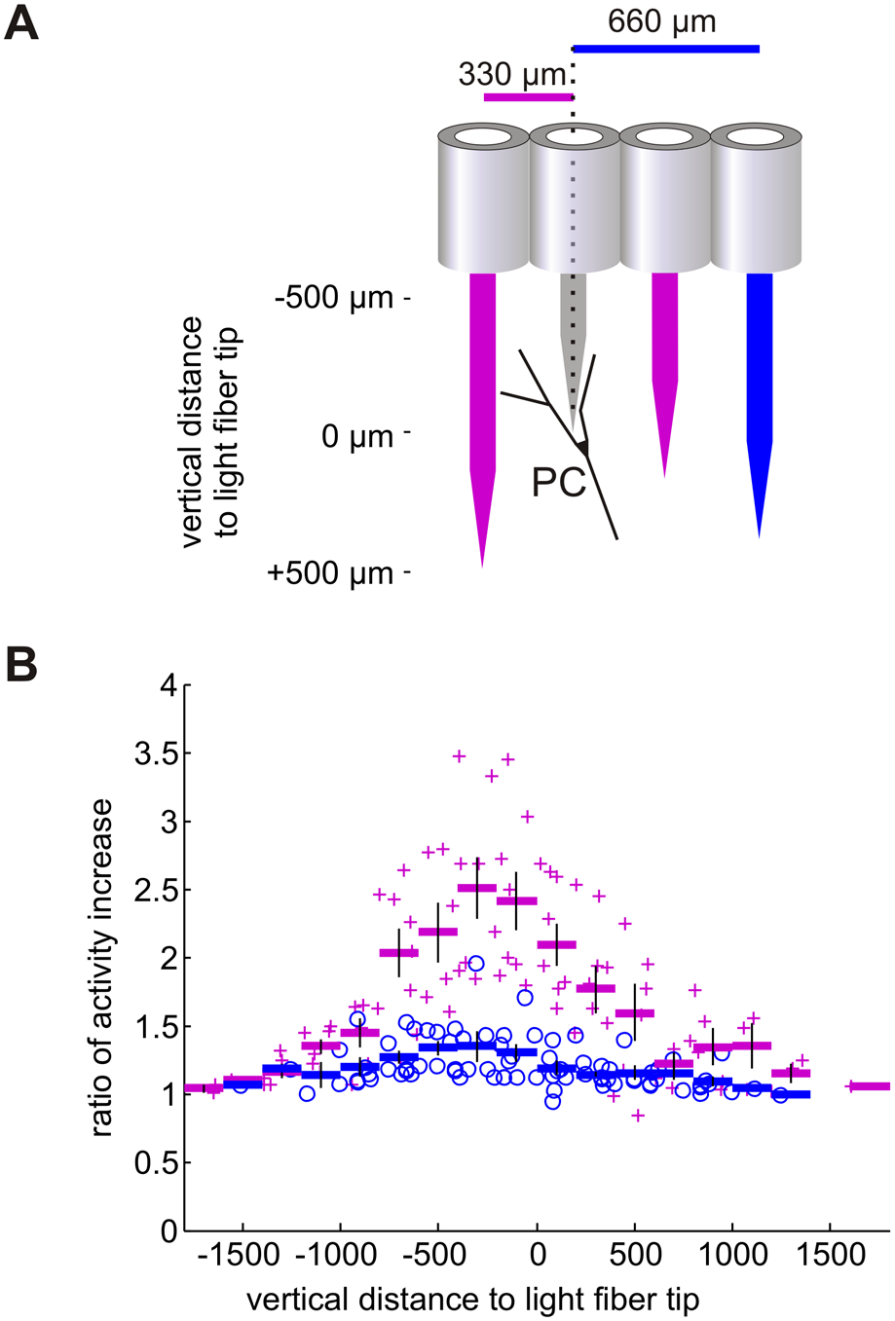
Light activation of PCs depends on the distance between the light source and the optogenetically targeted PC. (A) Schematic diagram of the relative distance of the light guide (red or blue) and the recording electrode (gray) around and along a PC. The light fiber was moved during the recording from responsive PCs in vertical direction relative to the recording electrode and 1000 ms pulses of light were delivered in various depths at 250 µm intervals. Laser power was varied between 0.5 and 5 mW, resulting in 0.125 to 1.25 mW in front of light fiber. The PC response to light was recorded, while the glass fiber was either in 330 µm horizontal distance relative to recording electrode (red fibers) or in 660 µm horizontal distance (blue fiber). (B) Diagram of the dependence of relative increase of PCs activity on the vertical distance of the light fiber tip. For each light guide position, activity during light application is expressed as multiples of spontaneous activity of the particular PC. Data points are aligned relative to the depth of the light fiber; negative distances correspond to positions of the light fiber above the electrodes. Activity increase elicited by light fiber positioned in neighboring guide tube (330 µm distance) are marked by red crosses (data from 10 PC recorded in 3 mice), rate increase caused by the light fiber positioned in the subsequent guide tube (660 µm horizontal distance) are marked by blue circles (data from 11 PC recorded in 3 mice). Averages are calculated for 200 µm intervals and plotted as horizontal bars, vertical lines indicate standard error of the mean. Note that strongest responses were elicited when the light fiber tip was up to 500 µm above the recorded PC.

To estimate the spatial domain of effective light emission and cell activation, we measured the light evoked PC activity as a function of distance between electrode and light fiber tip. For these measurements we first established a stable recording from a single PC or multiple PCs simultaneously. Responsive PCs showed a light-induced modulation of PC firing when light was applied through a neighboring glass fiber. Once a stable recording from a light-modulated PC was achieved, the depth position of the light fiber was changed relative to the recording electrodes and the light response of PC firing was measured. With this approach we quantified the modulation of PC simple spike rate either on neighboring light guide positions (330 µm horizontal distance of light fiber relative to recording electrode close to PC), or at 660 µm distance (Fig. 2A). At horizontal distances of 990 µm we failed to elicit significant modulation of cell activity with light. The light activation elicited from a neighboring glass fiber (330 µm horizontal distance) started to increase when the glass fiber was in cerebellar tissue 1500 µm above the recording site and reached a maximum when the tip of the glass fiber was approximately 250 µm above the electrode (red data points in Fig. 2B). When the light fiber was in 660 µm horizontal distance to the recorded neuron, the amount of light driven modulation was reduced (blue data points in Fig. 2B).

During electrode movement, we tested for the amount of light-driven modulation when a PC layer was approached. For this test, we drove the light guide to a similar depth as the electrode and adjusted light intensity to a level which caused strong modulation of PC activity temporally restricted to the time of light delivery. In some cells, the effect of strong light extended beyond the time interval of light application and caused a pause of activity after strong excitation (figure S3A). We occasionally observed also a reduction of an excitatory response during high light intensities in PCs, when directly compared to responses elicited with low light intensities in tgPcp2-cre mice (figure S3B). In such cases, the laser power was adjusted to obtain a strong and temporally precise activation of PC activity.

### Genetic targeting of ChR2 to different cerebellar cell types

To precisely characterize the effect of different cell types on the modulation of the intrinsic firing of PCs and to control PC firing specifically, we used different transgenic Cre lines in combination with double-floxed viruses expressing ChR2 (see Methods for details). For specific expression, the floxed virus was injected in the cerebellar vermis of tg-cre mice. Due to the PC specific promotor Pcp2, GABAergic interneuron specific promotor Gad2 and the GC specific promotor Gabra6, ChR2 expression was restricted to PCs, MLIs and GCs, respectively [26]–[28].

### Optogenetic activation of Purkinje cells in tgPcp2-cre mice

**Figure 3:**
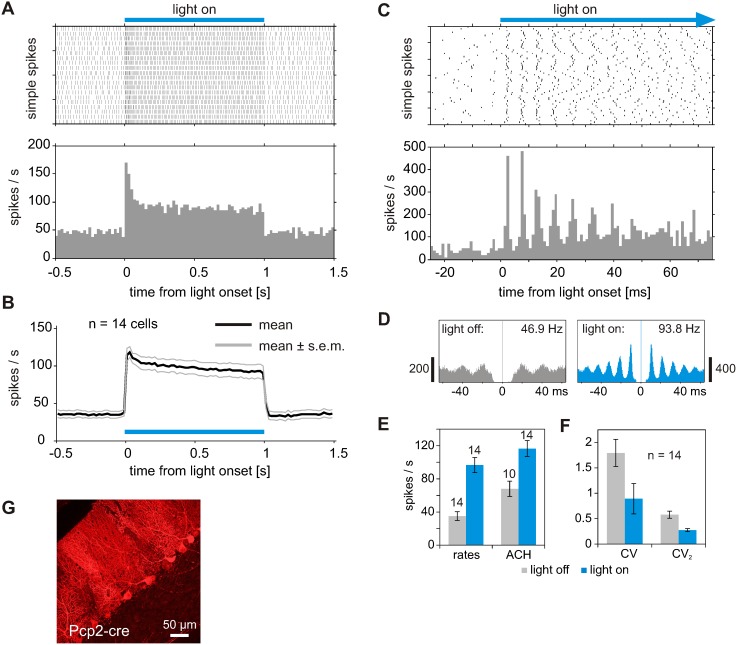
Activation of PC simple spikes during light activation of ChR2 specifically expressed in PCs. (A) Raster plot (top) and peri-stimulus time histogram (PSTH, below) during light application to PCs. The raster plot exemplifies a single cell response during 20 light applications; the PSTH shows the averaged single cell response during these 20 repetitions (bin width 20 ms). (B) Average response profile of 14 cells, recorded from 6 tgPcp2-cre mice expressing ChR2 selectively in PCs. Illumination with 473 nm stably increases simple spike firing of PCs. Laser power was set between 1 and 5 mW, resulting in 0.25 to 1.25 mW in front of light fiber. (C) Same cell as in A, but PC responses to 60 light pulses plotted at faster timescale. In higher temporal resolution, rhythmic firing in single simple spike trains and in the PSTH becomes evident (bin width 1 ms). Note that the simple spikes are reliably triggered in less than 6 ms after light onset. Subsequent simple spikes appear with little temporal jitter, which diminishes the rhythmic modulation in the PSTH over time. (D) To analyze rhythmic firing during light driven and spontaneous activity, autocorrelation histograms (ACHs) were calculated on simple spike trains from periods without (gray) and with light application (blue), respectively. The rhythmic modulation is discernible in the autocorrelation of spontaneous activity (46.9 Hz), but becomes more prominent during light driven activity (93.8 Hz, modal frequency obtained from Fourier transformed of ACH). (E) Average firing rates increase during light application from 35 to 97 spikes/sec (left, n = 14). When spectra were computed from ACHs, modal frequencies were dominant in all 14 spectra obtained from light driven activity, but only in 10 spectra obtained from spontaneous activity. Modal frequencies shifted in average from 68.0 Hz (n = 10) to 116.6 Hz (n = 14, 40 to 100 light applications per cell). (F) The coefficient of variation (CV) is strongly diminished during light application, also indicating that simple spike firing becomes more regular. Similar effect is seen when CV_2_ is calculated from adjacent inter spike intervals. (G) Parasagittal section of cerebellar cortex from Pcp2-cre mouse after virus injection. Red fluorescence indicates expression of hChR2(H134R)-mCherry specifically in PCs.

To investigate the functional effects of light-activation of different types of cerebellar neurons, we first recorded single PC activity during intrinsic modulation of PC firing by ChR2 (i.e. ChR2 is expressed in PCs). After recording from PCs was established at one or multiple electrodes, responses to light through the glass fiber was tested on all recorded signals. Activation of ChR2 in PCs causes the immediate and strong increase in the simple spike rate once light is applied (Fig. 3A). The simple spike rate remained at a constant high rate during continuous illumination of the cells. Complex spike rates in tgPcp2-cre mice were low in our experiments (0.23 Hz, n = 14), which was most likely related to isoflurane anesthesia. We did not see any influence of light application to the complex spikes (data not shown). As shown in Fig. 3A, the simple spike activity responds with a transient peak in the response rate, followed by a sustained response during continuous illumination. Such transient peaks were observed in 4 out of 14 cells, while the other cells showed mainly step-like enhancement in the firing rate during continuous illumination. The average peri-stimulus-time-histogram (PSTH) from 14 cells shows a plateau activity with only small fluctuations in the single cell responses (Fig. 3B, n = 14 cells from 6 mice). After light application, the simple spike activity decreased with short latency (16.4±5.3 ms, n = 14) to activity levels before light was applied (Fig. 3B).

The mean latency of the first peak in simple spike response after light onset was 3.9±0.45 ms, n = 14. After light onset, the majority of PCs (8 out of 14) show a rhythmic simple spike firing pattern which becomes obvious when data are plotted in high temporal resolution (Fig. 3C, same cell as in Fig. 3A). The sustained response of each PC showed a dominant frequency in their firing rate, which results in an oscillatory autocorrelation histogram (ACH) of the spike train during light application (Fig. 3D, right). During spontaneous activity, oscillatory modulation was weaker but still obvious in 10 out of 14 cells when dominant frequencies were obtained from the autocorrelations of the spike trains (example in Fig. 3D, left). In each cell, the dominant frequency obtained from the autocorrelation was higher than the spike rate calculated from the number of detected spikes (Fig. 3E). As previously noted by de Solages et al. [29], the discrepancy between spike rate and modal frequency is probably related to the occurrence of pauses in the firing of simple spikes. In the spontaneous firing (‘light off’), the mean of modal frequencies obtained from the autocorrelation is 72 Hz, and the light application shifts the simple spike frequency up to 115 Hz. For the spike rates, the corresponding mean values are 35 Hz (‘light off’) and 97 Hz (‘light on’) (Fig. 3E). With light application, the simple spike firing of the virus injected tgPcp2-cre mice becomes more regular, which is also reflected in the reduction of the coefficients of variation (CV and CV_2_, see Methods) during light application (Fig. 3F). When light intensity was varied, rhythmic patterns in simple spikes were usually observed even at lowest intensity levels emanating from the laser (0.5 mW corresponding 37.5 µW in front of the fiber, n = 4). Increase of light intensity failed to correlate with a higher regularity in the simple spike occurrence, quantified by CV and CV_2_ values (figure S3B). Similarly, there was no clear correlation between the response strength and the regularity in simple spikes when the response strength was varied by the distance of the light fiber from the recorded cell (as for data shown in Fig. 2). Although there was a clear tendency for a higher regularity in the simple spike timing during moderate activation of PCs in tgPcp2-cre mice (Fig. 3F), an increase in rhythmicity at higher light intensities seemed to be obscured by other irregularities in the simple spike response.

### Activation of inhibitory interneurons in Gad2-cre mice

**Figure 4:**
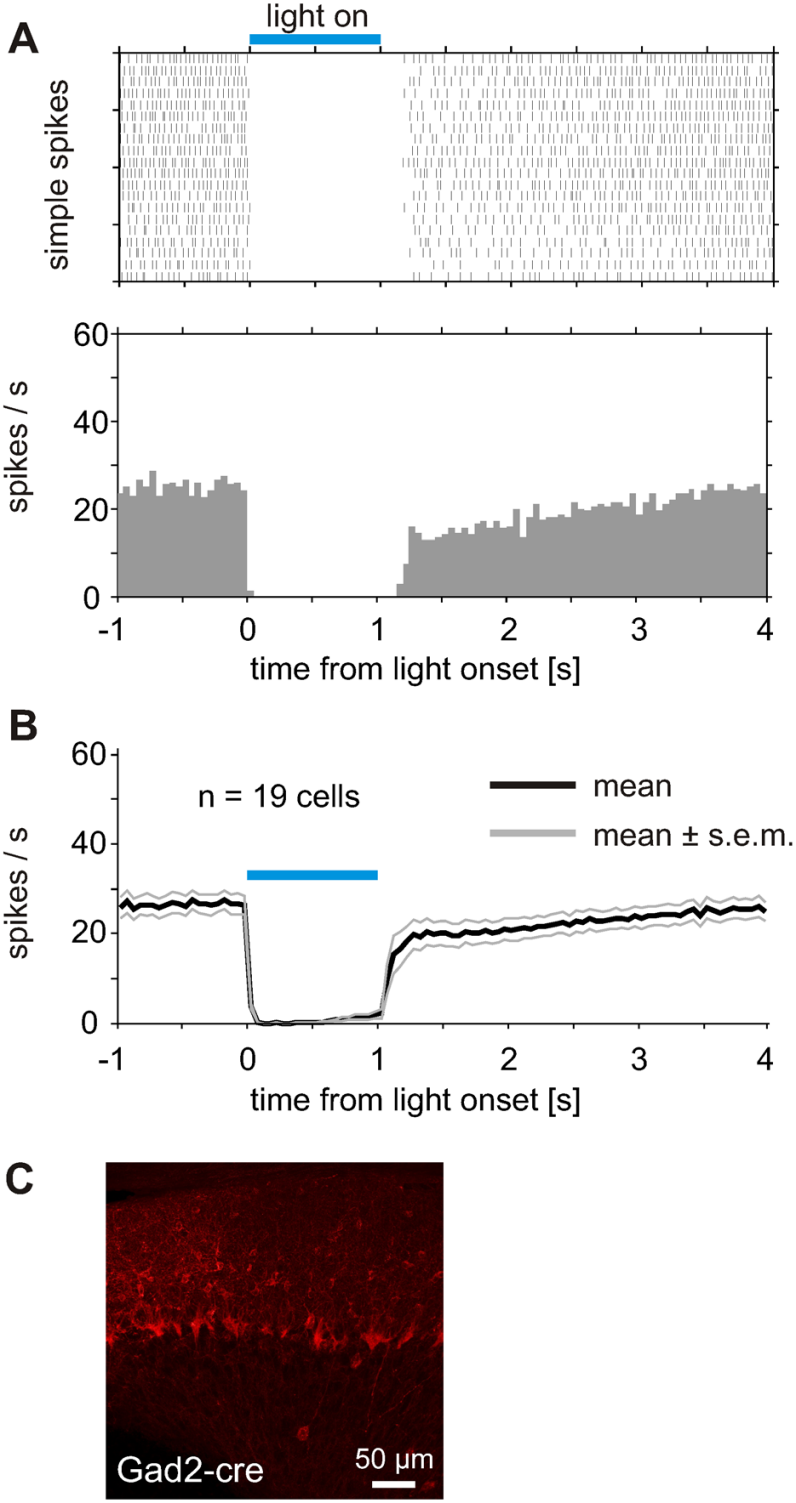
Inhibition of PC simple spikes during light activation of ChR2 specifically expressed in MLIs. (A) Complete inhibition of PC activity during light activation of MLIs in tgGad2-cre mice. As in figure 3, the raster plot is based on 20 light applications and the PSTH below shows the averaged response of the PC. (B) Average response from 19 cells responses with more than 3 sec interval between light applications (3 mice, 30 to 60 light applications per cell), showing a strong suppression of simple spike firing during a one second light application. Laser power was set between 2 and 5 mW resulting in 0.5 to 1.25 mW in front of light fiber. (C) Parasagittal section of cerebellar cortex from Gad2-cre mouse after virus injection. Red fluorescence indicates expression of hChR2(H134R)-mCherry specifically in inhibitory interneurons.

We next expressed and activated ChR2 specifically in MLIs using the Gad2t^m2(cre)zjh^/J mouse line [27]. Light activation of the MLIs completely blocked the simple spike activity of PCs in the vicinity of the light fiber. A typical suppression of simple spike activity is shown in Fig. 4A. The block of activity was complete as the simple spike rate was suppressed to zero for at least 500 ms. Only four out of 21 cells showed weak activity after 500 ms of light application, but even in those cells the simple spike rates were far below spontaneous activity measured before and after light activation. After light-induced inhibition of PC firing, simple spikes returned approximately 2.5 sec after light offset at the spontaneous rate measured before light application as indicated by the average activity of 19 cells recorded with more than 3 sec interval between light applications (Fig. 4B). The suppression of spontaneous simple spikes was transmitted with short latency as the last simple spike was observed only 3.6±0.4 ms after light onset (n = 21 cells from 3 mice). When a complete, 1000 ms long block of PC activity occurred during light activation of MLIs, the first spikes after light offset reappeared after 123.5±22.7 ms (n = 12 cells).

### Activation of granule cells in tgGabra6-cre mice

**Figure 5:**
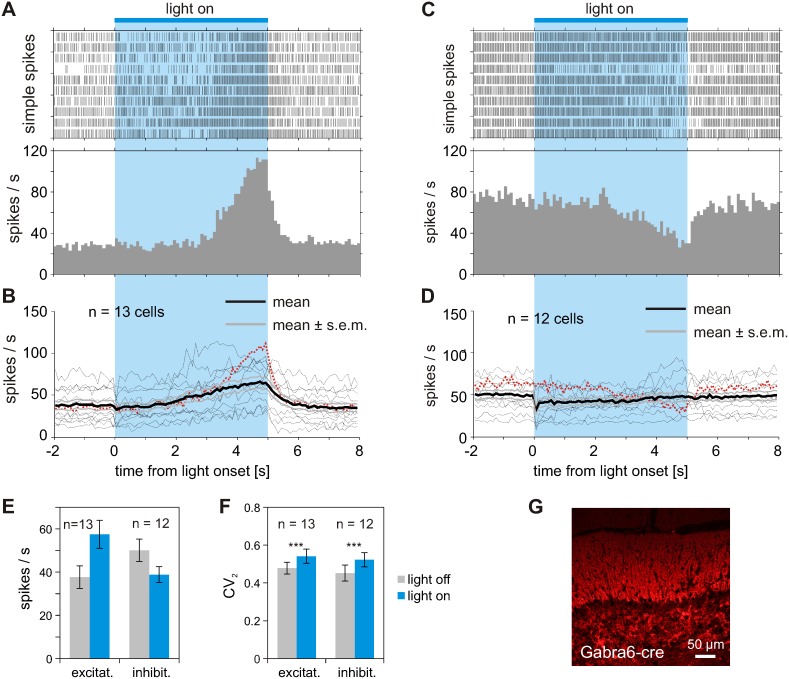
Multiple modes of modulation of PC simple spikes during light activation of ChR2 specifically expressed in GCs. Light activation of GCs results in inhibition and/or activation of PC simple spike activity. (A) Increase in PC spike rate during 5 sec light activation of GCs expressing ChR2. Raster plots and corresponding PSTH from 10 repetitions (bin size 100 ms). (B) Average response profile of 13 PCs showing increased activity during the time course of prolonged light application to GCs expressing ChR2 (12 to 20 light applications per cell). Bold black line indicates mean time course of rate modulation averaged across 13 PCs. Dotted red curve corresponds to example shown in (A) above. (C) Inhibition of PC simple spikes during 5 sec light activation of GCs expressing ChR2. Data from cells shown in A and C are recorded simultaneously with two different electrodes. (D) Average response profile of 12 cells showing decreased activity during 5 sec of light application to GCs. Data recorded from 3 mice, 12 to 20 light applications per cell. Dotted red curve corresponds to example shown in (C) above. Laser power was set to 10 mW, resulting in 2.5 mW in front of light fiber. (E) Mean increase of simple spike rate from 13 PCs showing excitation during light application and mean decrease of activity of 12 PCs showing inhibition during light application. (F) Regularity of simple spike firing was increased during light application. The increase in CV_2_ values was similar in both subgroups and therefore independent of the overall change in activity during light application. (G) Parasagittal section of cerebellar cortex from Gabra6-cre mouse after virus injection. Red fluorescence indicates expression of hChR2(H134R)-mCherry specifically in GC somas and parallel fibers crossing the dendritic trees of PCs in the molecular layer.

We next expressed and activated ChR2 specifically in GCs using the Tg(Gabra6-cre)B1Lfr/Mmucd mouse line [26]. We observed only weak changes in PC spike activity when activation of GCs was driven by light applications of less than 1000 ms. Therefore, we increased the time of light application to 5000 ms. During this prolonged period, either an increase (Fig. 5B, 13 out of 25 cells, 3 mice) or a decrease (Fig. 5D, 12 out of 25 cells, same 3 mice) in simple spike rates was measured. Compared to the results described above, the effect of GC activation on the activity of PCs had a slow time course, independent of whether the net effect was activating or inhibiting. The increase or decrease of activity could be observed in all three mice, and opposite effects were seen at different cells simultaneously. The examples of PC simple spikes rate changes shown in Fig. 5A and C were selected as they were recorded with different electrodes simultaneously. Both PCs show a pronounced change of activity during light application which was stronger than in the averaged responses plotted for activated or inhibited PCs in Fig. 5B and D, respectively.

Influence of GC stimulation on PC rhythmicity was not as obvious as during direct PC activation in Pcp2-cre mice. The spike rhythmicity was slightly diminished during light application in most cells and the fraction of PCs with clear side peaks in the autocorrelogram of simple spikes was lower during GC mediated modulation. This was similarly observed in PCs showing simple spike rate increase or decrease during light application. Our data from Gab6ra-cre mice showed higher variability in the ongoing activity and included occasional pauses for simple spikes. Therefore, to diminish the influence of such pauses we quantified the regularity in simple spike firing by calculating CV_2_ values (see Methods) from intervals during “light on” and “light off”, respectively. For 13 PC showing increased activity during GC stimulation, the CV_2_ increased significantly from 0.48±0.03 to 0.54±0.04, p<0.005, Wilcoxon signed rank test. Similarly, the average CV_2_ increased significantly for the 12 PC showing decreased activity during GC stimulation from 0.45±0.04 to 0.52±0.04, p<0.005 (Fig. 5E and F).

### Thermal effects on Purkinje cell firing

**Figure 6:**
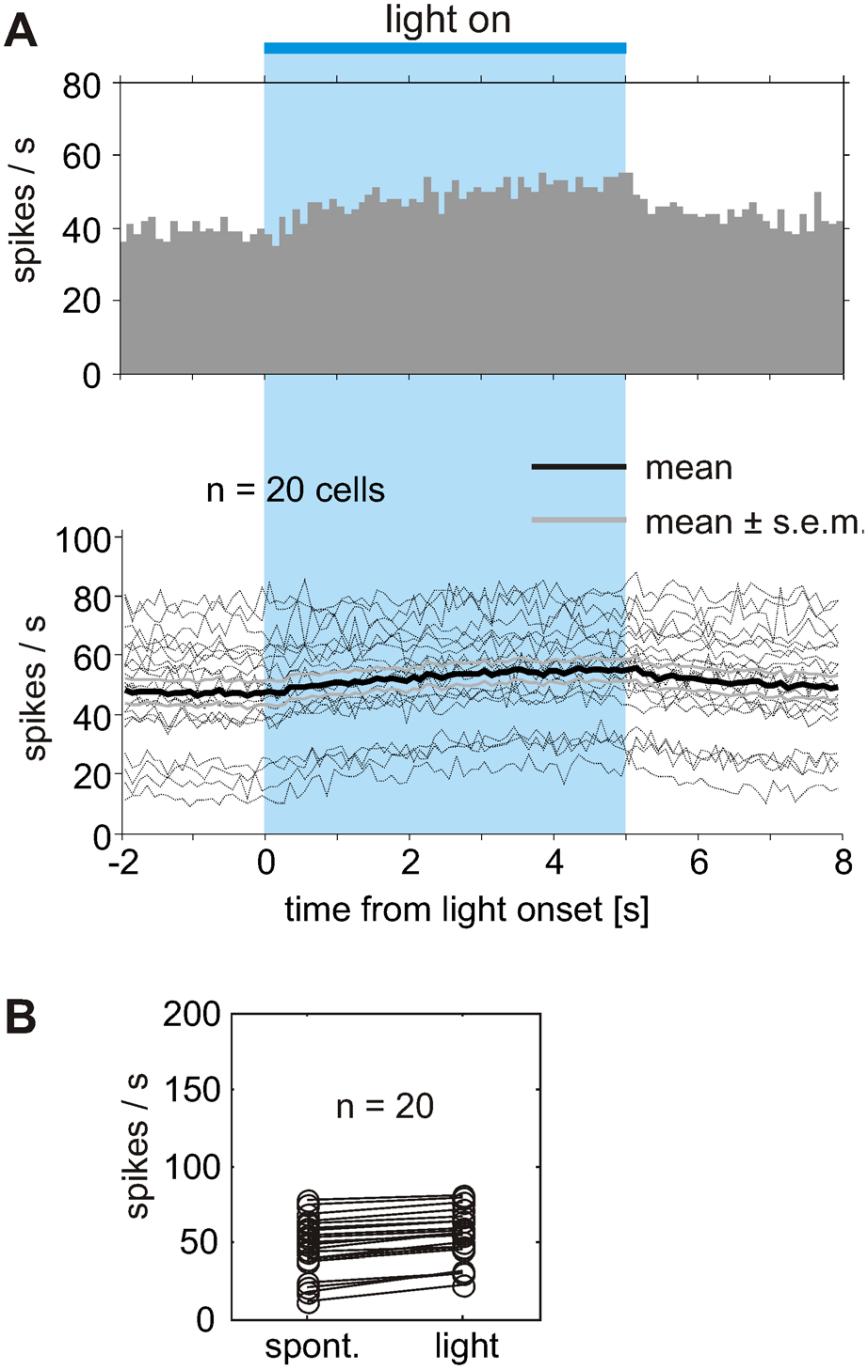
Thermal effect of light application on PC simple spike rates in tgGabra6-cre mice lacking ChR2 expression. PC simple spike rate responds to light application in tissue after expression of the tdTomato specifically in granule cells using AAV2/1.CAG-Flex.tdTomato. (A) Average response profile of 20 PCs showing increased activity during the time course of prolonged light application. Since no ChR2 is present, the observed increase in PC firing must be related to warming of the tissue surrounding the glass fiber. Light was delivered at maximum output power of the laser (20 mW), corresponding to approximately 5 mW measured in front of the tip. Bold black line indicates mean time course of rate modulation averaged across 20 PCs, 20 light applications per cell. (B) Increase in PC simple spike rate during light activation of cerebellar tissue expressing tdTomato in GC. Average activity increased from 47.5 to 54.4 Hz during light application.

The prolonged light application increased the total energy delivered to the tissue surrounding the light fiber, which might result in thermal effects on the surrounding neurons. To test for the influence of light application in normal tissue, which is not expressing ChR2, a viral marker (AAV2/1.CAG-Flex.tdTomato) was injected in the vermis of two tgGabra6-cre mice. During prolonged light activation for 5000 ms, an increase in simple spike firing rates could be elicited in all of the recorded PCs (Fig. 6). The average firing rate increased from 47.5±4.17 to 54.4±3.76 Hz (20 cells recorded in two mice). The light application with full output power of the laser (20 mW, corresponding to max. 3 mW emanating from the tip of the particular light fiber) led always to an increased simple spike rate. A decrease in spike frequency below base line levels was never observed during or after light application. The time constants of changes in PC activity during “light on” and “light off” were comparable (i.e. tau_light on_ = 2.30 and tau_light off_ = 2.20 sec), suggesting that increase and decrease of activity are driven by warming up and cooling down of the tissue surrounding the tip of the fiber. In contrast, the return of the mean GC driven activity after light application (Fig. 5B) reveals a time constant of 0.55 sec, indicating a faster change in activity than caused by cooling down of the tissue. We did not determine the time constant of the mean onset of GC driven activity, because single cells showed a very large variability in their onset responses (see Fig. 5B).

### Comparison of PC rate changes

**Figure 7:**
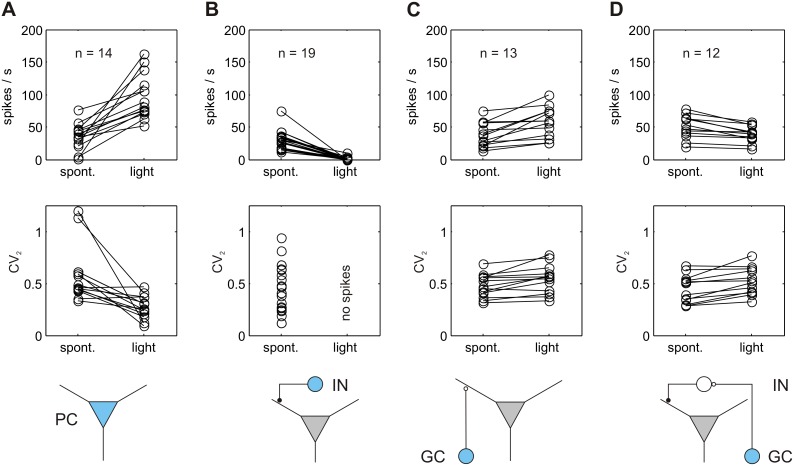
Comparison of PCs simple spike rate changes and spike regularity in response to light activation of PCs, MLIs and GCs. (A) Increase in PC simple spike rate during ChR2 mediated light activation of PC in Pcp2-cre mice (14 cells from 6 mice). The increase in spike rates is paralleled by an increase in spike regularity, quantified by lower CV_2_ values. (B) Inhibition of simple spikes in PCs during one second ChR2 mediated light activation of MLIs in Gad2-cre mice (19 cells from 3 mice). As PCs were silent during light application, CV_2_ values were not calculated. (C) Overall increase in simple spike rate during prolonged ChR2 mediated light activation (5 sec) of GCs in Gabra6-cre mice. In 13 out of 25 cells, the PC simple spike rate increased after GC light activation. The increase in spike rate is not accompanied by higher regularity in the simple spike occurrence as CV_2_ values increased (D) Overall decrease in simple spike rate during prolonged ChR2 mediated light activation (5 sec) of GCs in Gabra6-cre mice. The regularity of the spikes is reduced, as indicated by higher CV_2_ values. Twelve out of 25 cells showed a decrease in PC simple spike rate after GC light activation. The 25 cells were recorded from 3 mice. The schematic representations in the bottom row plots depict (in blue) the loci of ChR2-expression in the different mouse lines and indicate the possible synaptic interaction mediating the light activated cell activity onto the PCs. (PC: Purkinje cell, IN: inhibitory interneuron, GC: granule cell).

To summarize and compare the results of ChR2-mediated light activation of different cerebellar cell classes and their effects on PC activity, we plotted the changes in simple spike rates and the corresponding CV_2_ values of the analyzed PCs before and during light application. Expression and light-activation of ChR2 specifically in PCs (indicated in blue; Fig. 7A bottom) increased PC activity and is paralleled by an increase in spike regularity. The spontaneous rate of simple spikes more than doubled during light application (from 35.1±5.2 to 96.7±9.1 spikes/sec, Fig. 7A; see also Fig. 3E). Expression and light-activation of ChR2 specifically in MLIs, which make GABAergic synapses on either the dendritic tree or the soma of the PCs, causes a strong and sustained inhibition of all PCs recorded (Fig. 7B). GC mediated light-activation causes either an inhibitory or excitatory effect on PC firing. The increase in activity during long lasting light application (Fig. 7C, 13 out of 25 cells) is presumably related to direct activation of PCs via parallel fibers originating from the GC and projecting onto PCs (i.e. parallel fiber to PC synapse). Twelve out of 25 PCs showed an inhibition in simple spike firing after prolonged stimulation of GCs (Fig. 7D), which most likely involves the activation of projection of GC to MLIs followed by GABA release onto PCs. In both sub-populations, light activation of GCs results in reduced spike regularity, quantified by higher CV_2_ values.

## Discussion

Manipulation and precise control of neuronal activity is essential for the analysis of neuronal networks and, to date, has mainly relied on electrical microstimulation (e.g. [30], [31]). In contrast to slow pharmacological or other physical techniques (e.g. cooling or transcranial magnetic stimulation), electrical microstimulation has the advantage of controlling neuronal activity and cellular signals on a millisecond timescale. However, the precise control of specific neuronal pathways and cell-types is not possible with this technique. For example, co-activation of fibers during passage is unavoidable and large myelinated axons may be preferentially activated [32]–[34]. In addition, electrical stimulation requires voltage pulses that unavoidably generate an electrical artefact, which in general is larger than the neuronal signals of interest, meaning that the direct neuronal response to the electrical stimulus is difficult to determine.

The recent developments of optogenetic techniques to control neuronal circuits have overcome some of the limitations of electrical stimulation. Genetic targeting of different opsin variants allows for the cell-type specific expression and pathway specific control of neuronal circuits [14], [35]. The temporal kinetics for light-induced transmembrane currents can be chosen from a wide range of available opsins with time constants for channel closing ranging the low millisecond range (e.g. 4.0 ms; ChETA, [36]) through many minutes for step function opsins (see [37] for an overview). The dynamics of activation is not limited to light pulses: a sinusoidal modulation of light intensity is also capable of modulating cell activity [22] and activity can be enhanced by prolonged light application [16], [38]. Light exposure can generate an electrical voltage on metal surfaces, which might be especially confounding in local field potentials or in intracellular recordings (discussed in [39]). A low level of light intensity from a local source is therefore beneficial for reducing artefacts in the recording during light application. We used sustained activation with long light pulses at low intensities to additionally prevent interference of recurrent photoelectric artefacts with recording of neuronal signals.

In this paper we present the first results of a new method of light application through an optical fiber, which is assembled in a commercially available multi-electrode system (Thomas Recording, Giessen) and combines targeted light application with a proved system for extracellular multi-site recording. The usage of a customized optical fiber with a tapered tip allowed for independent positioning of optical fibers and electrodes into deep neuronal tissue. As the optical fiber has a geometrical shape similar to the standard microelectrodes for extra cellular recordings (Fig. S1), the optical fiber can be positioned in deep tissue with same spatial accuracy as the electrodes. Movement of the optical fibers had no discernible effect on recording stability of neighboring electrodes (tested with 330 µm radial distance). This technique is well suited to apply light to spatially restricted target areas in the brain.

The injection of a double floxed virus for ChR2 expression in tgPcp2-cre mice enabled us to reliably activate PCs with light. The widespread expression of the ChR2 in the vermis allowed us to directly measure the spatial extent of the light emission from the tip of our light fibers. The area of activation extended approximately 1500 µm around the tip of the light fiber (Fig. 2), with an optimal activation 250 to 500 µm in front of the tip. The activation of PCs located above the stimulation site might be either related to the omnidirectional radiation of the fiber tip (Fig. S2B) or by reflection of the light in tissue.

### Comparison to other fiberoptic and LED techniques for combined optogentic activation and recording in the cerebellum

Different approaches have been used in recent studies to apply light to cerebellar tissue in *in* *vivo* applications. Light from a laser was either targeted on the cerebellar surface by means of a light guide [16], which was positioned approximately 8 cm above the cerebellum of rats to illuminate a surface spot with 1 mm diameter. With this approach light intensity at the tissue surface was set to 50 mW/mm^2^ and ChR2-expressing PCs were reliably activated. Another study by Witter et al. [40] applied blue light to the entire cerebellum by means of strong LEDs positioned around the skull of a mouse. The device allowed for graded stimulation capable to activate PCs on every stimulation pulse. This whole field light stimulation was chosen to elicit rebound responses in deep cerebellar nuclei, which required a strong and widespread activation of PCs throughout the cerebellum. The same study used awake mice to elucidate motor responses after focal illumination of the cerebellar surface at lobule V and VI of the anterior vermis, for which a 400 µm optic fiber was implanted in a small craniotomy. Witter et al. [40] estimated that this approach was able to activate between 150 and 400 PCs in the most superficial Purkinje layer, which was sufficient to evoke a motor response due to synchronous disinhibition of the deep cerebellar nuclei after cessation of light driven PC activity. These approaches were limited to the superficial layers of the cerebellum, as the light is strongly scattered in tissue [37], [41].

Here, we explored the effectivity of the usage of customized light fibers with a tapered tip. This technique allows for focal stimulation of deep targets and enables perturbation of cerebellar cortical and subcortical function in a spatially restricted manner [42]. Comparable local application might be possible with techniques combining light fiber and recording electrode in a single device as in optrodes, either as “dual-pronged” assemblies of a light fiber and a conventional electrode, or as coaxial optrodes [24] and even multichannel optetrodes [23]. These assemblies have still larger tip diameters than single conventional microelectrodes, which may increase tissue damage, especially in the cerebellar cortex of mice. Insertion of a double tipped (dual-pronged) optrode seems unfavorable for experiments where extracellular activity is recorded simultaneously as the damage to the tissue might be extensive in the cerebellum (for comparison, see damage in primate cortex documented in [24]). For the same reason, we did not insert acutely a flat-cleaved optical fiber into mouse cerebellum. Most likely this approach would have generated too much pressure on the tissue and would have disturbed the recording at neighboring electrodes. The optical fibers used in the current study had tapered ends with less than 40 µm diameter, which were conically ground to allow for gentle positioning in the tissue.

The tapered tips used in our study produced an omnidirectional radiation of light (Fig. S2) to avoid damage to the tissue [38]. Omnidirectional radiation spatially minimizes zones of high radiation, whereas for flat-cleaved tips emission is given by the numerical aperture of the fiber resulting in a cone shaped beam form with a less steep decline in radiation density [37]. The broader distribution of light from the tapered tip minimizes the area where the risk of an unwanted depolarization block exists. Such an inhibition of activity in ChR2-expressing neurons has been described previously [38], [43] and was observed in a few cases with our tapered tip glass fibers at high light densities and strong expression levels of ChR2. Additional to the reduction of simple spike activity at high light densities a transient block of PC activity was observed after strong excitation (Fig. S3). Such suppression resembles a rebound hyperpolarization triggered by activation of calcium-dependent potassium conductances [44] and could therefore be an indication of strong calcium influx triggered by ChR2 excitation.

### Light-induced tissue heating

A negative side effect of light application comprises an increase of neuronal activity due to tissue warming. We tested for this effect by injecting AAV2 expressing tdTomato into the vermis. As a result, a reliable increase in simple spike rate was observed in PCs when 3 mW light was applied for 5 sec to the tissue (Fig. 6). When we tested for temperature effects on the simple spike rate of PCs in slice recordings, an increase of 15 Hz per degree C was observed when temperature was varied between 30 and 34°C (unpublished observations). Accordingly, we estimate that the temperature increase at the recording sites was well below 0.5°C. The effect of tissue heating has been discussed in Yizhar et al. [37] and the authors emphasize the need for opsin negative controls to estimate the effect of heating. From their calculations they estimate a temperature increase of 0.38°C at a distance of 500 µm in front of the tip when 5 mW light is emitted from the tip. Thus, tissue heating is a common side effect of light application and has to be considered especially in cases where light application is spatially separated from the targeted area.

### Targeted control of Purkinje cells, molecular layer interneurons and granule cells through ChR2

The injection of double floxed virus for ChR2 expression in tgPcp2-cre mice enabled us to selectively activate PCs by light. The simple spike rate of PCs increased immediately after light on and remained at an increased level during the time course of light application (Fig. 3A and B), even when light was delivered for several seconds. Comparable results have been described previously during light activation of ChR2 under the control of a PC-specific promotor L7 [16]. In our study, the temporal pattern of simple spikes showed an increased regularity during light application in all cells tested (n = 14). The higher regularity is quantified by the reduced coefficient of variation (CV and CV_2_). It is also manifested in oscillatory patterns in the auto-coincidence histograms calculated from simple spikes during light on compared to light off (Fig. 3C–E). We retrieved the dominant frequency of the simple spikes from the frequency spectrum of these oscillations in the auto-coincidence histograms [29], as such patterns are a clear indication of a repetitive simple spike activity with a constant inter spike interval. The increased rhythmicity pattern in the occurrence of simple spike after light on and during prolonged light activation is probably an outcome of the continuous transmembrane currents through activated ChR2.

The dominant frequencies calculated from the auto-coincidence histograms are higher than the mean rates as spike counts and are affected by the presence of pauses in simple spike firing [45]. Similar differences between dominant frequencies and spike rates have been described by de Solages et al. [29].

We failed to reveal a clear dose-response relationship between light intensity and simple spike regularity. The occurrence of oscillatory simple spikes is most prominent in weak to moderate responses but becomes less regular when higher light intensities are applied to responsive PCs in tgPcp2-cre mice. The weakening of oscillatory modulation might also be related to an increase in rebound hyperpolarization after strong calcium influx.

In the Gad2-cre mouse line, the suppression of PC simple spikes during activation of MLIs was fast and reliable and blocked the simple spikes for the time course of the light activation (Fig. 4). At light onset, the simple spike firing either ceased immediately with no further spikes after light onset or one simple spike occurred within a few milliseconds after light onset. Therefore, the inhibitory synaptic transmission from the stellate or basket cells seems to have an immediate, strong inhibitory effect on the PC simple spike firing [46]. A similar effect has been described in recent work by Heiney et al. [47], where PC simple spikes were inhibited via optogenetic activation of MLIs, capable of eliciting discrete orofacial and eyelid movements.

In contrast to the fast activation and inhibition of PCs via light-induced depolarization of PCs or MLIs, light-induced activation of GCs causes a slow modulation of PC firing. Light pulses of 1000 ms used in other studies were not sufficient to modulate PC firing. Therefore, we had to use higher light powers (2.5 mW in front of light fiber) and a five times longer light application to modulate PC firing. Since GCs only make single synapses with PCs via the parallel fiber (PF) many PFs have to be activated to induce modulatory effects. GC activation caused either an increase or a decrease in PC simple spike firing. The increase in PC firing most likely involves the direct activation of PF-PC synapses, while the inhibition may involve the activation of MLIs synapsing onto PCs by GCs [48]. *In vivo* patch clamp recordings showed that GCs are under tonic inhibitory influence [49]. The GC layer may have a functional role in temporal structuring mossy fiber input, as sensory input through mossy fibers evokes short burst in GCs after punctate stimulation ([50] reviewed in [51]).

The slow effect of GC stimulation is counterintuitive to the finding that synapses from ascending GC axons provide an excitatory input to local PCs [52]–[54]. These ascending GC axons should contribute to a fast excitation of PCs after GC stimulation. However, we never observed such fast activation at the beginning of GC stimulation. One can speculate whether the optogenetic stimulation has to be spatially more focused to areas lying directly under the recorded PC to activate ascending GC axons. This might require an angled approach of the light guide to the PC layer such that a neighboring light fiber with 330 µm distance reaches the GC layer at an appropriate position. Such approaches will be included in our future experiments. The surprising long latency of more than one second for the activation of PCs after onset of GC stimulation might be an outcome of competing excitatory and inhibitory effects. From 25 PCs recorded during 5 sec GC stimulation, almost equal numbers showed either an increase or a decrease in overall activity. On the level of single PCs, there might be a competition of direct activation of PCs via parallel fibers and inhibition mediated by MLIs. The superposition of such competing inputs may determine whether the PCs are more or less active during GS stimulation and may prevent a fast response of PCs to GC stimulation. In comparison to recent studies by Witter et al. [40] and Chaumont et al. [43] we failed to reliably affect complex spikes with light application. This might be related to the fact that we used isoflurane anesthesia during our recordings which is known to affect various voltage-gated ion channels [55], whereas Witter et al. used ketamine/xylazine and Chaumont et al. used urethane anesthesia. The overall low rates in complex spikes may have obscured any effect on the complex spikes in our experiments.

In conclusion, we developed a method to control the distance between an optical fiber relative to a recording electrode using the Eckhorn matrix system for simultaneous optogenetic manipulation and recordings of neuronal activity. Because of the adjustable positioning of the light fiber relative to the electrodes, the system allows to precisely activate light-activated proteins and signaling cascades in a cellular volume domain of approximately 1500×660×660 µm^3^. Within this activation domain we were able to control different cell-types of the cerebellar cortex to modulate PC firing. We found that light-activation of PCs and MLIs switch PCs on and off with milliseconds time-precision, whereas activation of GCs slowly modulates firing of PC depending on which cellular pathway is activated.

## Materials and Methods

### Ethics statement

All experiments were in strict accordance to the European Communities Council Directive RL 2010/63/EC, and the NIH guidelines for care and use of animals for experimental procedures and have been approved by the local government ethics committee (LANUV NRW, (Landesamt für Umwelt, Natur und Verbraucherschutz Nordrhein Westfalen, Düsseldorf) permit number 87-51.04.2010.A090). All surgical procedures and all electrophysiological recordings were performed under isoflurane anesthesia and all efforts were made to minimize suffering and number of animals.

### Mouse strains

TgPcp2-cre mice (stock #004146 B6.129-Tg(Pcp2-cre)2Mpin/J; [28]), C57BL/6J (stock #000664) and Gad2-cre (stock #010802; Gad2^tm2(cre)Zjh^/J; [27]) were purchased from the Jackson Laboratory. TgGabra6-cre mice (stock #000196-UCD; B6; D2-Tg(Gabra6-cre)B1Lfr/Mmucd; [26]) were purchased from Mutant Mouse Regional Resource Center (MMRRC). All animals used for the experiments were bred and raised in the animal facility of the Department of Zoology and Neurobiology.

### Adeno associated viruses and injection

To transduce different cerebellar cell types selectively, we injected the pAAV-Ef1a-DIO-hChR2(H134R)-mCherry-WPRE (AAV2/9.EF1a.dflox.hChR2(H134R)-mCherry.WPRE.hGH virus construct obtained from Penn Vector Core, University of Pennsylvania, Addgene plasmid 20297, P.I. Deisseroth) in the cerebellum. This virus contains a double-floxed inverted open reading frame (DIO) under the control of the EF1-α promoter and is inserted in all cells in the target area of the virus injection. Due to the double floxed opsin sequence, ChR2 was only expressed in cells expressing Cre recombinase from the different transgenic mouse lines. Adeno associated virus expressing double floxed ChR2 was injected into the cerebellar vermis of tgPCP2-cre mice where ChR2 is expressed under the control of the Cre/Lox system in only Purkinje cells [28], in Gad2-cre mice where the injection of floxed ChR2 is expressed exclusively in GABAergic inhibitory interneurons, i.e. stellate and basket cells [27] and in tgGabra6-cre mice where expression of ChR2 is only in granule cells [26].

For virus injection the mouse was anesthetized with 1–2% isoflurane in oxygen delivered from a precision vaporizer (E-Z Anasthesia, Euthanex Corp, Palmer, PA, USA) and placed in a stereotactic frame (SR-6M, Narishige, Tokyo, Japan). A sagittal incision along the midline was made to expose the cranium, and a burr hole was drilled 6.0 mm posterior to the bregma, targeting the vermal part of lobule V and VI. The tip of a glass micropipette (tip diameter ∼20 µm) attached to a syringe was lowered into the vermis. Several injections were administered at depths between 2000 and 500 µm until 3 to 5 µl of virus were injected. After each injection the pipette was left in place for 2 min before it was retracted to a higher position. After the final injection the pipette was left in place for additional 5 min to secure proper reception of the virus in the tissue. The scalp incision was sutured. The mouse was maintained under a 37°C heater and under observation until recovery from the anesthesia, before returning to standard cages. Mice were maintained for 14 days before performing electrophysiological experiments.

### Histology

Animals were anesthetized and perfused intracardially with 4% paraformaldehyde in PBS. Brains were removed, fixed for 1 h at 4°C, and then cryoprotected overnight by incubating in 30% sucrose in PBS. Samples were embedded in optimal cutting temperature medium and immediately frozen in dry ice. Then, 20 µm cryostat sections were collected, air-dried at room temperature on gelatin-coated slides. Images were acquired with a Leica TCS SP5 confocal laser scanning microscope (Leica DMI6000 B, Wetzlar, Germany) using a 20X/0.7NA and 40X/1.1NA objective.

### Electrophysiological recording

For electrophysiological recordings, the mouse was anesthetized and placed in the stereotactic frame as described for virus injection. The burr hole from the virus injection was exposed again and the craniotomy was enlarged to a diameter of 1.5 to 2 mm by use of a dental drill. The dura was carefully removed. Extracellular activity was recorded with a multi-electrode system (Eckhorn system, Thomas Recording, Giessen, Germany), and extracellular signals of up to six electrodes (impedance, 2–3 MOhm at 1 kHz; Thomas Recording) were simultaneously amplified and filtered (band-pass, 0.1–8 kHz) with a multichannel signal conditioner (CyerAmp380, Axon Instruments, Union City, CA, USA). All signals were sampled with 32 kHz via an A/D converter (NI PCI-6259 multifunction data acquisition board, National Instruments, Austin, TX, USA) controlled by custom-made software implemented in Matlab (MathWorks, Natick, MA, USA). All signals were stored on a PC for offline analysis conducted in Matlab.

### Light application

For light application into the cerebellum a light conducting glass fiber was mounted in the multi-electrode system. A graded index glass fiber with 125 µm diameter cladding and numerical aperture NA = 0.275 (GIF625, ThorLabs, Newton, NJ, USA) was stripped to remove the acrylate coating. The front end of the fiber was commercially heat-pulled and ground (Thomas Recordings, Giessen, Germany) to match the tip geometry of the standard platinum-tungsten electrodes (Fig. S1). With an outer diameter of 125 µm, the stripped fiber fits in the 330 µm outer diameter guide tube of the multi-electrode system. The fiber was equipped with a rubber tube and a pulling string similar to the recording electrodes allowing the positioning of the glass fiber in axial direction with similar precision as the electrodes [20]. The upper end of the fiber protruded from the multi-electrode system and was guided through a hole in the lid of the Eckhorn system to a fiber connector. See (text S1) for the required adaptations to house the glass fiber in the multi-electrode system.

A FC/PC patch cable (ThorLabs) served to connect to a 473 nm diode pumped solid-state (DPSS) laser (BCL-473-020-M, CristaLaser, Reno, NV, USA) with adjustable output power of 20 mW maximum. The laser was gated by a TTL signal provided by the NI multifunction board, which was driven by the Matlab software controlling the data acquisition. When the maximum power of 20 mW was emitted by the laser, a total power in the range of 1.5 to 5 mW was measured in front of the customized fiber tip with a laser power meter (Model 407A, Spectra Physics, Darmstadt, Germany), depending on the particular light fiber used in the experiment. The spatial distribution of light emitted at the pulled and ground tip of the fiber was more strongly dispersed than the light emitted from a fiber with a flat, polished end (Fig. S2).

Laser light was applied as pulses of 1000 or 5000 ms duration and an inter-pulse interval of 2000 to 5000 ms. The power of the laser was set to the range from 20 to 0.5 mW, yielding an output power at the tip in the range of 5 to 0.0375 mW, depending on the transmittance of the light fiber.

### Data analysis

In the off-line data analysis, single unit action potentials were detected with custom-made software implemented in Matlab. A spike train was considered to originate from a PC when simple and complex spikes were observed in the same signal of a single electrode. Simple spikes occurred spontaneously with a frequency between 20 and 150 Hz, whereas complex spikes were encountered less frequently with a rate of below one spike per sec. Complex spikes were characterized by multiple wavelets following a strong depolarizing spike. The origin of both spikes from a single PC was proved by the presence of the simple spike pause (“climbing fiber pause”) after each complex spike [2], [25]. Simple spike rates during light application were compared with simple spike rates obtained during spontaneous activity. To quantify the spike train regularity, coefficients of variation (CV) of interspike-intervals (ISIs) [CV = stdev (ISI)/mean(ISI)] were calculated. Additionally, the coefficients of variation for adjacent intervals CV_2_ of ISIs [CV_2_ = 2 | ISI_n+1_–ISI_n_ | /(ISI_n_+ISI_n+1_)] [56] were calculated. An average of CV_2_ over n estimates the intrinsic variability of a spike train, nearly independent of slow variations in average rate. Error bars denote SEM except when indicated otherwise. Exponential fit of activity after stimulation onset and offset was made with IgorPro (WaveMetrics, Portland, OR, USA).

## Supporting Information

Figure S1 **Tip geometry of recording electrode and optical stimulation fiber.** (A) Standard platinum/tungsten in quartz microelectrode (Thomas Recording) with 80 µm shaft diameter and conically pulled and ground tip. The metal core of the electrode appears darker than the surrounding quartz glass (B) Bare glass fiber with stripped coating leaving a shaft diameter of 125 µm inclusive cladding (GIF625, ThorLabs, Graded-Index Multimode Fiber, 0.275 NA). The tip was commercially heat-pulled and ground (Thomas Recording) to match the geometry of the standard recording electrode.
(TIF)

Figure S2 **Light emission of a tapered light guide and flat-ended glass fiber.** (A) Light emission from perpendicularly cleaved and polished glass fiber (GIF625 ThorLabs, numerical aperture 0.275). The maximum light intensity is along the fiber axis with a steep fall off to the sides according to the numerical aperture of the glass. (B) Light emission from similar fiber (GIF625, ThorLabs, NA 0.275) with customized tip (Thomas Recording). Emission is almost circular with similar intensity in all directions, giving a better chance to activate cells at surrounding electrodes in the multi-electrode setup. Both fibers are positioned in air above scale paper having a 1 mm grid. Black broken lines are superimposed to indicate position of the fibers.
(TIF)

Figure S3 **Decrease of PC simple spike rates during strong light application.** PC simple spike rate increases during weak light application (laser power set to 0.5 mW resulting to 0.0375 mW measured in front of the tip) compared to spontaneous simple spike rate, but is reduced during strong light application (20 mW at laser corresponding 1.5 mW in front of fiber tip). (A) PSTHs from PC responding to 1 sec light application in tgPcp2-cre mouse after injection of floxed ChR2. Activation during light is gradually decreased with increasing light intensities. Note the sustained block of spontaneous spikes after offset of strong light pulses, as indicated by arrows. (B) Average response rates from four PCs recorded simultaneously. Recording was performed with four individually positioned electrodes, with their tips less than 1000 µm apart from tip of light guide (horizontal distance: 330 µm, axial distance between 270 µm above to 850 µm below light guide). Data shown in (A) are from electrode E7. (C) Regularity of simple spikes is increased for low light intensities as CV_2_ values drop below reference values, but become more irregular for higher light levels.
(TIF)

Text S1 **Assembling optical stimulation fiber into the multi-electrode system.**
(DOCX)
