# Supplementary figures and images for: Optogenetic Modulation and Multi-Electrode Analysis of Cerebellar Networks In Vivo
Source: PLoS One. 2014 Aug 21;9(8):e105589. doi: 10.1371/journal.pone.0105589 (PMC4140813; doi:10.1371/journal.pone.0105589)

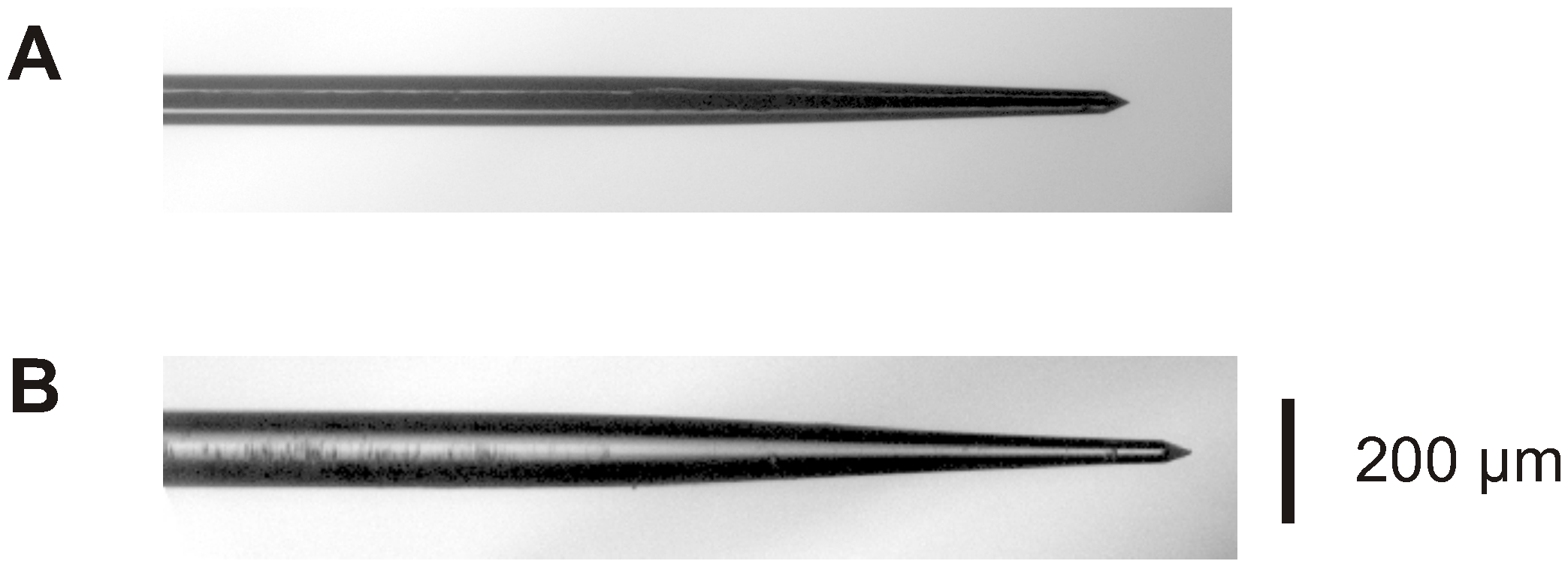

Supplement: Figure S1 — Tip geometry of recording electrode and optical stimulation fiber. (A) Standard platinum/tungsten in quartz microelectrode (Thomas Recording) with 80 µm shaft diameter and conically pulled and ground tip. The metal core of the electrode appears darker than the surrounding quartz glass (B) Bare glass fiber with stripped coating leaving a shaft diameter of 125 µm inclusive cladding (GIF625, ThorLabs, Graded-Index Multimode Fiber, 0.275 NA). The tip was commercially heat-pulled and ground (Thomas Recording) to match the geometry of the standard recording electrode. (TIF) [file pone.0105589.s001.tif]

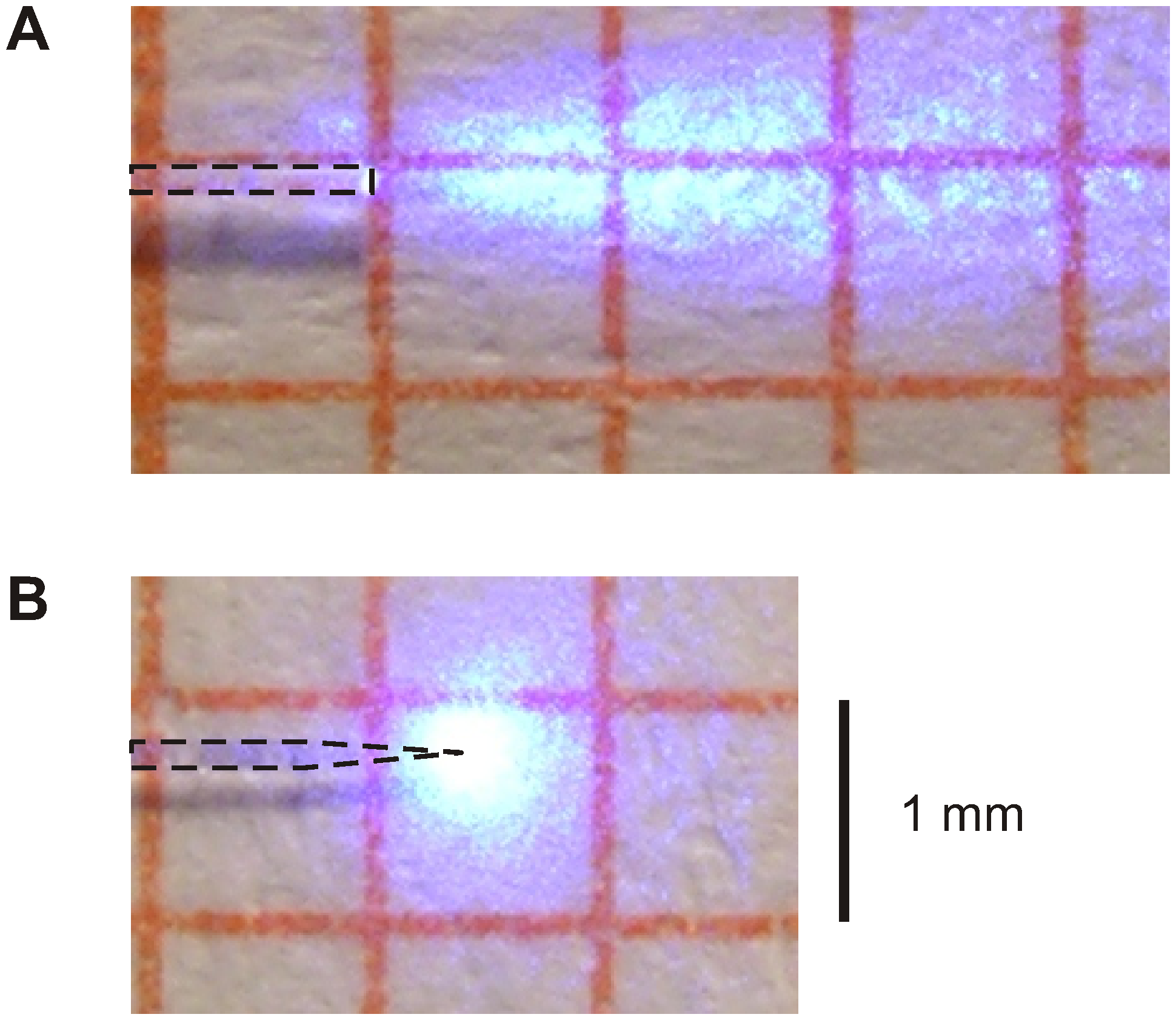

Supplement: Figure S2 — Light emission of a tapered light guide and flat-ended glass fiber. (A) Light emission from perpendicularly cleaved and polished glass fiber (GIF625 ThorLabs, numerical aperture 0.275). The maximum light intensity is along the fiber axis with a steep fall off to the sides according to the numerical aperture of the glass. (B) Light emission from similar fiber (GIF625, ThorLabs, NA 0.275) with customized tip (Thomas Recording). Emission is almost circular with similar intensity in all directions, giving a better chance to activate cells at surrounding electrodes in the multi-electrode setup. Both fibers are positioned in air above scale paper having a 1 mm grid. Black broken lines are superimposed to indicate position of the fibers. (TIF) [file pone.0105589.s002.tif]

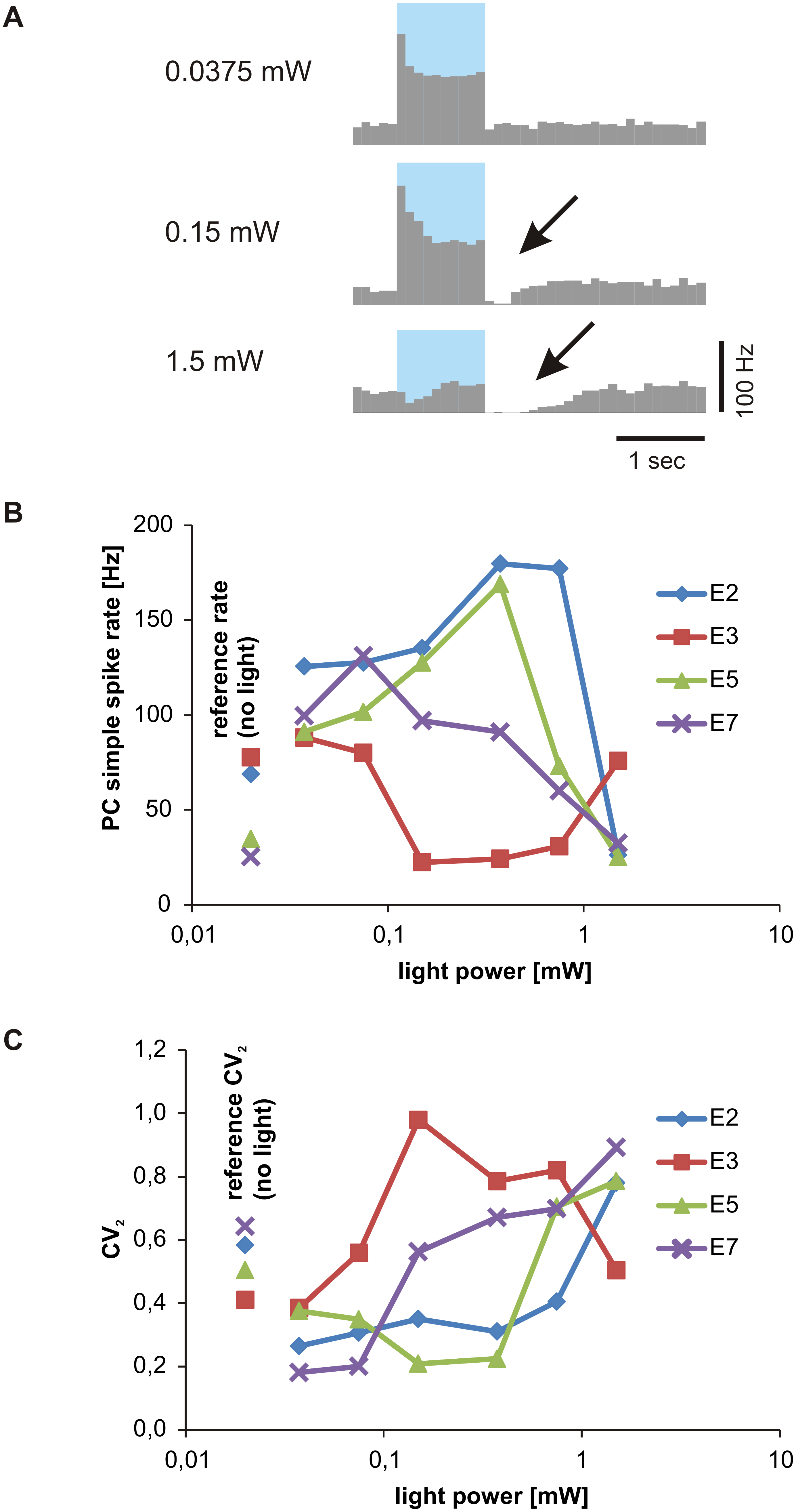

Supplement: Figure S3 — Decrease of PC simple spike rates during strong light application. PC simple spike rate increases during weak light application (laser power set to 0.5 mW resulting to 0.0375 mW measured in front of the tip) compared to spontaneous simple spike rate, but is reduced during strong light application (20 mW at laser corresponding 1.5 mW in front of fiber tip). (A) PSTHs from PC responding to 1 sec light application in tgPcp2-cre mouse after injection of floxed ChR2. Activation during light is gradually decreased with increasing light intensities. Note the sustained block of spontaneous spikes after offset of strong light pulses, as indicated by arrows. (B) Average response rates from four PCs recorded simultaneously. Recording was performed with four individually positioned electrodes, with their tips less than 1000 µm apart from tip of light guide (horizontal distance: 330 µm, axial distance between 270 µm above to 850 µm below light guide). Data shown in (A) are from electrode E7. (C) Regularity of simple spikes is increased for low light intensities as CV2 values drop below reference values, but become more irregular for higher light levels. (TIF) [file pone.0105589.s003.tif]
