# Supplementary material for: Optogenetic Modulation and Multi-Electrode Analysis of Cerebellar Networks In Vivo
Source: PLoS One. 2014 Aug 21;9(8):e105589. doi: 10.1371/journal.pone.0105589 (PMC4140813; doi:10.1371/journal.pone.0105589)
Supplement: Text S1 — Assembling optical stimulation fiber into the multi-electrode system. (DOCX) [file pone.0105589.s004.docx]

**Assembling optical stimulation fiber into the multi-electrode system**

The Eckhorn multi-electrode system (Thomas Recording, Giessen, Germany) is designed for the controlled positioning of electrodes or other thin and delicate fibers into brain tissue. Therefore, the integration of an optical fiber into the multi-electrode system was pursued to allow for precise depth positioning of the fiber independent of the electrodes.

The raw glass fiber (GIF625, Graded-Index, Ø62.5 µm Core/Ø125 µm Cladding, Multimode Fiber, 0.275 NA, ThorLabs, Newton, NJ, USA) was cut in pieces of 260 mm length and both ends of the fiber were stripped to remove the acrylate coating according to the guidelines provided by ThorLabs (www.ThorLabs.com). The front end of the fiber to be used for manufacturing the tip was stripped at a length of 90 mm to allow for the heating and pulling procedure with subsequent grinding of the tip. The tip was manufactured and customized by Thomas Recording, Giessen, Germany. In a first step, the tip was pulled in a computer controlled pulling chamber so that a slim conical tip was achieved. Subsequently, the tip was ground on a diamond disk to shape a taper at the very end of the tip. The fiber was rotated around its axis during the grinding to assure a precisely centered taper. The ground surface of the tip is the predominant area of light emission when a light source is connected to the other end of the fiber.

The removal of the acrylate coating allowed for the proper fit of the fiber (125 µm outer diameter with cladding) in the multi-electrode guide tubes. The upper end of the fiber was stripped at a length of 20 mm to insert it into a fiber connector (ST fiber connector, Farnell, Oberhaching, Germany) which was fixed to a ST/FC adapter (Farnell). It was avoided to permanently fixate the fiber in the connector with epoxy to allow an easier removal of the fiber from the system. Instead, the fiber was manually pushed in the ferule of the connector until it had contact to the fiber of the patch cable. The physical contact of the fiber with the patch cable was paralleled by an increase of light emission at the tip of the fiber.

The transmission of the whole fiber system was checked before each experiment by measuring the output power with a laser power meter (Model 407A, Spectra Physics, Darmstadt, Germany) positioned with its absorbing surface less than two millimeters in front of the tip. With laser output set to 20 mW, a total power measured in front of the tip was in the range between 1.5 to 5 mW, i.e. a maximum of 25% of the laser power was emitted at the tip of the fiber.
